# Towards a New Biomarker for Diabetic Retinopathy: Exploring RBP3 Structure and Retinoids Binding for Functional Imaging of Eyes In Vivo

**DOI:** 10.3390/ijms24054408

**Published:** 2023-02-23

**Authors:** Vineeta Kaushik, Luca Gessa, Nelam Kumar, Humberto Fernandes

**Affiliations:** 1 Institute of Physical Chemistry, Polish Academy of Sciences, 01-224 Warsaw, Poland; 2 Integrated Structural Biology Group, International Centre for Translational Eye Research, Institute of Physical Chemistry, Polish Academy of Sciences, 01-224 Warsaw, Poland

**Keywords:** diabetic retinopathy (DR), retinol binding protein 3 (RBP3), two-photon excitation fluorescence (TPEF), diagnostics

## Abstract

Diabetic retinopathy (DR) is a severe disease with a growing number of afflicted patients, which places a heavy burden on society, both socially and financially. While there are treatments available, they are not always effective and are usually administered when the disease is already at a developed stage with visible clinical manifestation. However, homeostasis at a molecular level is disrupted before visible signs of the disease are evident. Thus, there has been a constant search for effective biomarkers that could signal the onset of DR. There is evidence that early detection and prompt disease control are effective in preventing or slowing DR progression. Here, we review some of the molecular changes that occur before clinical manifestations are observable. As a possible new biomarker, we focus on retinol binding protein 3 (RBP3). We argue that it displays unique features that make it a very good biomarker for non-invasive, early-stage DR detection. Linking chemistry to biological function and focusing on new developments in eye imaging and two-photon technology, we describe a new potential diagnostic tool that would allow rapid and effective quantification of RBP3 in the retina. Moreover, this tool would also be useful in the future to monitor therapeutic effectiveness if levels of RBP3 are elevated by DR treatments.

## 1. Introduction

**Figure 1:**
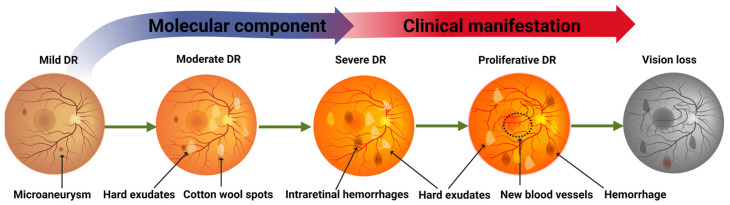
DR progression from mild to proliferative to vision loss. Also represented are the starting points and the increasing progression of the molecular and clinical components of DR with typical lesions.

Diabetic retinopathy (DR), a highly prevalent consequence of diabetes, is a progressive disease and major cause of vision loss [1,2]. It is frustrating that DR is still occurring in larger numbers despite the availability of preventable measures. Moreover, as diabetes itself is reaching an epidemic level, DR numbers will continue to rise [3]. This prospect is particularly alarming, as the risk of losing vision in a person with diabetes is 25-fold higher than in someone not afflicted by diabetes [3,4]. The reasons for this rise are numerous and complex. First, there is a time gap between the molecular evolution of the disease and the onset of the clinical observations (i.e., DR remains largely asymptomatic until late in the pathology [1,5]) (Figure 1). Regarding treatment, laser photocoagulation and pharmacological intervention are possible options, but they are usually administered at later stages and are not always efficient in stopping the progression of the disease. Therefore, the best DR countermeasure is still strict control of diet to keep blood glucose levels low. One undisputable bottleneck preventing clinicians from breaking the cycle is the need for an early diagnostic tool that can identify individuals who need more frequent screening, guidance for lifestyle changes, or preventative medical intervention [2]. Indeed, it has been shown that effective screening when associated with prompt intervention diminishes vision-threatening DR (VTDR) [1,6,7,8].

### 1.1. Diabetic Retinopathy

The number of people suffering from DR is rising (calculated to reach 191 million by 2030 [4,9]), and expected to double in the next 25 years. Despite improvements in the condition of diabetic patients (and thus a lower percentage of DR cases per total diabetic patients), diagnosis and treatment of DR is as relevant as ever, considering the historic incidence of diabetes that has elevated the number of people suffering from the disease to epidemic status. The current diagnostic methodology is only semi-quantitative and categorizes different fundus observations into non-proliferative retinopathy, diabetic macular edema, or proliferative diabetic retinopathy. This leaves approximately 50% of diabetes patients diagnosed as having no retinopathy [10]. Therefore, there is a need for discovery of molecular biomarkers that can be quantified and serve to signal the onset of the disease and its progression. This is particularly important as it is now recognized that known risk factors (such as longer duration of diabetes, poorly controlled blood-glucose level, poor blood pressure, and dyslipidemia) are not fail-safe indicators as they are not always present in patients that develop DR, and some patients who do display these risk factors do not evolve to DR status nevertheless [11].

DR is one of the consequences of diabetes, resulting from high glucose levels in the blood due to the lack of, or insensitivity to, insulin [1]. Clinically, DR is divided into two branches, proliferative and non-proliferative disease. In the proliferative stage, the most distinctive feature of DR is new vessel formation. Non-proliferative diabetic retinopathy (NPDR) is normally graded as mild, moderate, or severe (the stage preceding proliferative DR). Although DR is a direct consequence of diabetes, there is a delay between the diabetes diagnosis and the appearance of DR.

**Figure 2:**
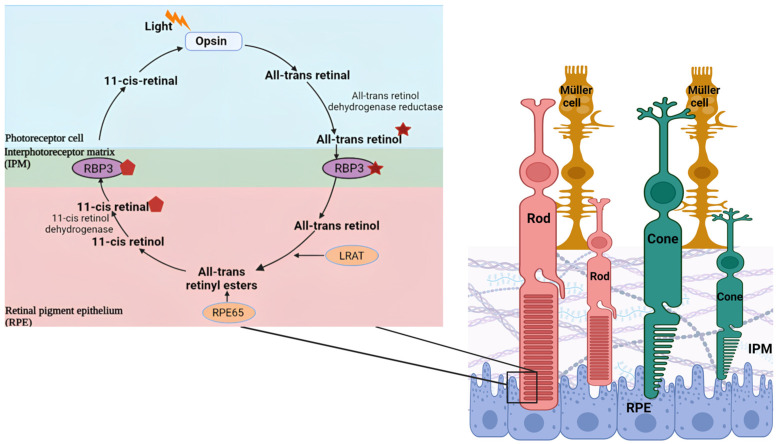
Simplified composition of the retina with RPE, photoreceptors, Muller cells, and IPM represented. Insert: Visual cycle.

It has now been established by electroretinography (in patients and animal models), contrast sensitivity, and color-vision tests that neuroretinal function is impaired in DR before the onset of vascular lesions (which can be seen in fundus imaging, for example) [2,5,9,12,13,14,15,16,17,18,19,20,21,22,23,24,25]. However, these functional tests are not always practical. Hence, molecular techniques for detecting biomarkers are very important and are currently being developed, and the list of targets has been expanding [11,26]. At the same time, however, we should be developing imaging techniques that allow for non-invasive and immediate readout of abnormalities in retinal function to anticipate DR clinical manifestations [13]. Several vitreous biomarkers (mainly angiogenic and anti-angiogenic) factors have already been shown to vary according to the absence or presence of DR [3,11], but we want to push the timeline of detection of DR-driven changes even earlier, as well as localize the indicators closer to the photoreceptors where DR ultimately plays a more profound, damaging role. Therefore, we have focused on examining retinol binding protein 3 (RBP3), also known as Interphotoreceptor Retinoid-Binding Protein (IRBP), in the interphotoreceptor matrix (IPM). DR is known to affect various retinal cells, especially several types of retinal ganglion cells (RGCs) [27,28]. However, we decided to focus on/near photoreceptors, as they are the most abundant cell types in the retina and provide a much more homogenous sample. It is important to identify “clean” biomarkers, whose origins are well-defined and only altered in a single disease state. RBP3 may be just such a biomarker, as it is secreted exclusively by photosensitive tissues, resides in the IPM (the space between photoreceptors and Retinal Pigment Epithelium (RPE) cells), and plays an important role in the visual cycle, shuttling retinoids between the two cell types [29,30,31,32,33,34,35,36] (Figure 2).

**Figure 3:**
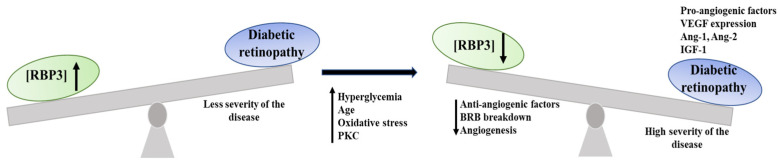
Homeostasis disruption upon DR progression, with decreased levels of RPB3 in the IPM with advances in the severity of DR.

RBP3 has been implicated in several studies of DR [14,31,37], the latest of which clearly indicates a relationship between RBP3 levels in the retina (and vitreous) and the severity and progression of DR [30,37] (Figure 3). These observations are further supported by the fact that overexpression (or “injections” of externally produced RBP3) in rodents inhibited DR-related events [29,37]. The molecular mechanism underlying such observations seems to relate to the binding of the RBP3 antagonist to the glucose transporter 1 GLUT1 (present at outer segments and cell bodies of photoreceptors); as well as to RBP3 binding to the vascular endothelial growth factor (VEGF) receptor and inhibiting tyrosine phosphorylation [29,37]. The likelihood that such interactions decrease the retinal concentration of RBP3 link it to the progression of the disease [29,37].

### 1.2. Eye Imaging

In eye diagnostics, there is a delicate balance between how much one needs to “see” and how much radiation must be delivered to enable the observations. Fluorescent imaging has long been used in medicine for diagnostics and labelling purposes. Despite being an “open window”, the eye is also a very fragile organ that harbors the photoreceptors that are extremely sensitive to photodamage. Depending on the degree of tissue penetration needed, short wavelength light with high energy may be necessary, and thus intrinsically damaging, while it is still possible that it does not even penetrate to the desired depth due to being absorbed, for example, by the lens.

Current DR imaging diagnostics are qualitative and rely on subjective interpretation. They depend on structural imaging with visualization of the retinal vasculature, either without dyes (fundus photography) or with the introduction of dyes (fluorescein angiography); or on changes in the thickness of the retinal layers (optical coherence tomography). These methods, in addition to their intrinsic diagnostic limitations, also require the participation of very specialized practitioners, at the very least to interpret the data; and the resultant costs, associated with the high prevalence of DR, are difficult to sustain even in healthy countries [1,3,38,39,40]. Currently, TPEF is also expensive to operate and requires skilled technicians, but the readout of our proposed tools would be a distinct number that can be easily checked against a table by potentially anyone. This approach is in contrast to the interpretation of complex images or plots, as with optical coherence tomography (OCT) and electroretinogram (ERG), that require trained personnel to be decoded. Moreover, compared to the other techniques, TPEF detects not only structural changes but also metabolic ones and does so at a high contrast and subcellular spatial resolution. Efficient tracking of the disease also involves increasingly frequent screening that further increases total costs; and since only 8–10% of diabetic patients develop VTDR, this is not easily attained [1]. Alternatively, according to recent data indicating that retinal neurodegeneration (in the absence of microvascular disease) is also involved in DR, functional inefficiencies can be measured with electroretinograms, although those are even more costly than assays that track vasculature [1,24,25]. To overcome this burden and more efficiently use the resources, we propose the use of two-photon excitation (TPE) fluorescence (TPEF) to quantify RBP3 levels and acquire a quantifiable biomarker that can assist with early diagnosis of DR. Early diagnosis is crucial, as the best line of defense against DR is, so far, early intervention in lifestyle and diet changes, followed by laser surgery, vitrectomies, and anti-VEGF treatment.

**Figure 4:**
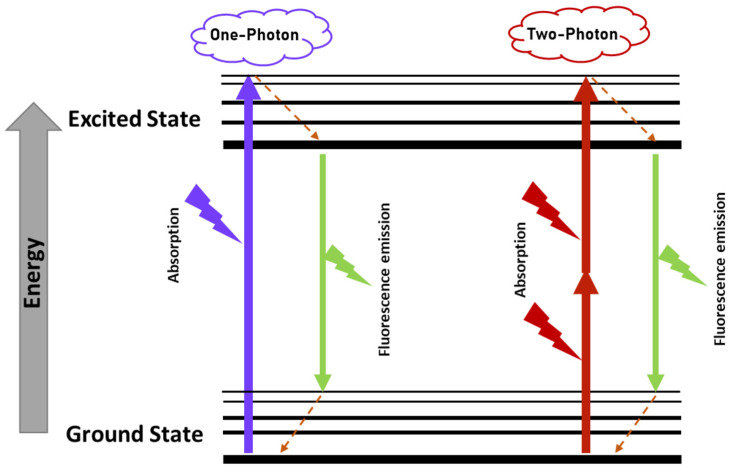
Jablonski diagram for one- and two-photon excitation fluorescence.

Two-photon excitation fluorescence microscopy is a variant of optical microscopy that offers several advantages compared to, for example, confocal, light-sheet, and super-resolution microscopy [41,42]. TPEF uses two near-infrared photons to achieve excitation (Figure 4), minimizing the autofluorescence generated by the imaged tissues, reducing photodamage, and achieving a greater penetrative power [42,43].

This method was first proposed in 1990 [44] and has since been evaluated in various medical applications such as protein quantifications in cancer settings [45,46,47], as well as Alzheimer’s [43], drug release [48,49], and ophthalmologic contexts [50]. Here, we suggest an additional application of TPEF. We propose that RBP3 levels can be measured in patients’ eyes and quantified as a proxy for DR status. In situ protein quantification has already been addressed using two-photon microscopy, allowing precise human NAD(P)H:quinone oxidoreductase (hNQO1) activity monitoring precisely in human colon tissue [47], and monitoring of β-Galactosidase (β-gal), carboxylesterase (CES), and hNQO1 enzyme activities in gastric cancers [45]. TPEF has also been used in fluorescence lifetime imaging microscopy to identify a gradient of human serum albumin (HSA) from normal connective tissue to the tumor tissue, with HSA being labelled with the two-photon probe squaraine (SD) [46]. Fluorescent lifetime, in simple terms, uses the time that a fluorophore stays in the excited state, offering yet another fingerprint feature of fluorophores, which is particularly important when several endogenous fluorophores with largely overlapping spectra are present [42]. We propose that the selectivity can be achieved by using probes that react selectively with retinol-binding proteins and that it will be possible to specify RBP3 by narrowing the measurements to the IPM [51,52,53], where RBP3 is by far the most abundant RBP protein.

## 2. Diabetic Retinopathy

It is important to distinguish between the two DR progressions; one is clinical, while the other is molecular. For the former, there are known, traceable cues that indicate disease progression. Whereas, for the latter, several biomarkers have been proposed, but so far these suffer from limitations that cannot be overcome that preclude their becoming truly universal indicators of early signs of the disease.

### 2.1. Clinical Progression

DR is a progressive disease with clinical manifestation occurring in the retina vasculature before patients detect any vision issues. Initial microaneurysms, and hemorrhages, lead to hypoxia, which leads to neovascularization and then to loss of visual acuity [1]. The primary stages, known as non-proliferative diabetic retinopathy (NPDR), are monitored for signs of aggravation, but treatment is reserved for the later stages, when changes in the pathology trajectory are harder to achieve [2], after signs of neovascularization have begun (proliferative DR (PDR)). Further complication is associated with the presence of hard exudates (yellow or whitish deposits of leaked lipids), which is known as diabetic macular edema (DMO) and typically located at the center of the macula [1].

DR is clinically detected when small circular “dark lesions” become apparent in fundus photographs and swelling of the macular retina is detected. The lesions originate from microaneurysms (thought to be in part due to loss of pericytes) or “dot/blot” hemorrhages or light exudates. Microaneurysms (i.e., focal dilatations) can be located within the inner nuclear layer and can lead to tiny (dot) or larger (blot) hemorrhages due to the rupture of weakened capillaries (i.e., aneurysms). Superficial layers of the retina can also suffer hemorrhages, which usually cause blood to track along nerve fibre bundles. Blood vessel leakage also leads to exudates (fat deposits, such as lipoproteins), which present as yellow spots typically within the retina [1]. These changes can also lead to early signs of ischemia, which are usually not seen in fundus photographs but become evident in fluorescein angiograms.

### 2.2. Molecular Component

Hyperglycemia is the root cause of DR, but oxidative stress, inflammation, activation of protein kinase C (PKC), accumulation of advanced glycation end products (AGEs), and dysregulation of the polyol, renin-angiotensin, and hexosamine pathways all contribute to dysfunction of the vascular endothelium [1,5,26,54,55,56,57,58,59].

AGEs are the consequence of surges in non-enzymatic glycosylation of proteins due to increased levels of glucose in the circulation that become irreversible during the progression of the disease [1,60,61,62], resulting in pericyte apoptosis, neovascularization, and increased inflammation [1]; as well as capillary occlusion and ischemia [5]. High glucose levels also dysregulate glucose metabolism in general and in the polyol pathways (which convert glucose into sorbitol and, later, into fructose) in particular. The excessive production of fructose diminishes the NADPH pool, resulting in an increased ratio of oxidized to reduced glutathione and, in turn, oxidative stress [1]. The excess of glycolytic intermediates also results in de novo synthesis of the protein kinase C (PKC)-activator diacylglycerol (DAG) [1]. PKC is known to cause several vascular dysregulations [1].

Oxidative stress [9,63] causes vascular lesions such as capillary degeneration. The link of oxidative stress to DR [29,64,65,66,67,68,69,70,71] is made abundantly clear by the fact that the overexpression of antioxidant enzymes (such as superoxide dismutase) and the administration of antioxidants inhibit such lesions [29,69,70,72]. The ischemia/hypoxia observed in the retina as a consequence of vascular dysregulation results in an increased expression of VEGF via activation of hypoxia-inducible factor 1 (HIF-1) [5,73] and possibly also via elevation of phospholipase A2 (PLA2) [5,74]. Other angiogenic factors may play a role in the escalation of the problem [5,75,76], but VEGF is currently the only target for DR treatments.

In addition to vascular dysregulation, inflammation is a core component of DR pathogenesis, with leukostasis being an important contributor beginning in the early stages of DR [5]. Notably, increases in cytokines (such as tumor necrosis factor alpha (TNF-α) and several interleukins) and in endothelial cell adhesion molecules directly correlate with the severity of DR [5]. The inflammation pathways are also related to the elevated expression of chemokines, such as monocyte chemotactic protein-1 (MCP-1) [5].

An important aspect of DR that makes early diagnosis particularly important, is the observation of metabolic memory [3]. With dogs, 2.5 years of poor glycemic control followed by 2.5 years of good glycemic control results in a prognosis that is as bad as that for five years of impaired control, indicating that some metabolic memory is maintained (and likely promoted) in DR progression. The DR complications observed in the retinal vasculature do not seem to generate any reduction in outer nuclear layer (ONL) thickness, measured by OCT, indicating no loss of photoreceptors in such a large animal [29]. The factors involved in such memory are not fully elucidated but likely involve AGEs and epigenetic markers. Thus, the sooner correct glycemic control is achieved, the lower the possibility of long-term problems. There are some indications that histone deacetylase inhibitors improve diabetic nephropathy [3], but early diagnosis and timely glycemic control are a better option for the patient, preventing but not reversing the DR-related consequences. Oxidative stress can elevate AGEs and epigenetic markers, and thus, antioxidants add another layer of defense to limit the progression of DR when initially detected [3].

In addition to the consequences and triggers that oxidative stress causes in the vasculature, it is now known that the primary site of oxidative stress is the avascular outer retina [2]. Some in vitro studies also implicate RPE cells in the generation of oxidative stress in the DR context [29,77], seemingly through the sub-part of the visual cycle that occurs in RPE cells, as an RPE65 inhibitor (retinylamine) inhibits the typical DR-associated increase of superoxide in the retina [29,78]. Such oxidative stress originates in RPE cells; in addition to disrupting RPE cell junctions and barrier integrity [29,79], it also affects other retinal cells, particularly photoreceptors [29]. Photoreceptors themselves are generators of oxidative stress in more significant amounts than other retinal cells, such as endothelial cells, pericytes, and Muller cells [29,68]. Several studies have now shown that both RPE cells and photoreceptors show several metabolic abnormalities, including oxidative stress, during the clinical silence phase of DR progression [2,68,78,80,81,82,83,84,85,86,87,88,89,90,91,92,93,94,95,96,97,98,99,100], and this has been observed in retinal cryosections stained with a reactive oxygen species-specific probe [2,68,101]. It should be noted that photoreceptors and IPM, in contrast to RPE and Muller cells for example, have fewer antioxidant systems due to the different expression of antioxidant systems including phase II enzymes [102,103,104,105].

Importantly, treating such oxidative stress early on can alleviate histopathology in diabetic models and restore vision [2]. In previous work, antioxidant treatment of diabetic animals restored the nondiabetic open/close phenotype of cyclic nucleotide channels and rod L-type calcium channels (LTCCs) in response to dark/light adaptation [2,91,106,107,108]; and also remedied the diabetic-induced dysfunction in interphotoreceptor matrix hydration changes, with water mobility observed between dark and light-adapted retinas [2,66,109]. Another study, initially using a cell line but later validated in ex vivo human retinal explants and in a mouse animal model, indicated that the introduction of Simvastatin offers neuroprotection to the photoreceptor by boosting RBP3 protein production [110], thereby protecting the photoreceptors from oxidative stress induced by all-trans retinal (atRAL). atRAL is a major source of drusen components and known to induce oxidative stress on retinal cells [110,111,112]. Another report indicated that tetramethylpyrazine upregulates RBP3 expression [30,112]. Importantly, RBP3 contains several cysteines, many of which are free thiols, allowing RBP3 to act potentially as a free radical scavenger [102].

### 2.3. Biomarkers

The pursuit of biomarkers, particularly circulating biomarkers, must take into account their origins and their true relevance to disease as a diagnostic option. RBP3 is only expressed in photoreceptor cells (with some trace mRNA expression being shown in the pineal glands) [30,113]. Thus, it is a good potential candidate because alterations to its presence should be solely due to alteration in the tissue of interest. Moreover, invasive biomarker collection is potentially unpleasant for the patients, and in the case of DR and the retina it is not possible, as biopsies cannot be performed on the retina. Samples could be taken from the vitreous and serum, but the RBP3 levels are progressively much lower there, and such collection would raise additional issues on collection practice and potentially on its storage. Thus, being able to measure RBP3 in the retina non-invasively has multiple advantages.

RBP3 resides in the interphotoreceptor matrix and, in the retina, it is solely produced by photoreceptors; variation in its content has a clear and direct relation with the severity of DR, making it an excellent candidate as an early biomarker for DR. Moreover, current methodology has been focused on biomarkers of inflammation and angiogenesis, and not on oxidative stress factors. This is not surprising as oxidative stress biomarkers are notoriously difficult to measure and often unreliable [3]. However, RBP3 may represent an exception as a window into the oxidative stress status in DR and a “clean” biomarker.

Generally, biomarker quantification/detection is best performed through biopsies, but in the case of DR, the eye’s retina is not suitable for such a procedure. The second-best method involves the collection of vitreous fluid from patients undergoing vitreoretinal surgery, but such a procedure is usually performed when the clinical signs of DR are already present, and as we will argue below, much later than the original molecular imbalances [31]. We propose another approach that aims to validate the quantification of RBP3 as an early biomarker of DR, as RBP3 is produced in photoreceptors, inversely correlates with the severity of DR, and can be monitored in a non-invasive, biopsies-like, procedure. The detection of DR biomarkers before the onset of ophthalmoscopic signs (microaneurysms, hemorrhages, and cotton-wool spots) could allow the initiation of treatment even while functional vision remains unperturbed [12]. RBP3, measured in situ and non-invasive, also removes concerns related to sample collection and storage [3].

### 2.4. Non-Invasive Tracing of Disease Manifestations

Fundus photography and OCT, mentioned above, are important non-invasive tools for the diagnosis of DR, allowing changes or abnormalities in OCT to be observed before detectable changes in the fundoscopic examination [3]. In addition to these two methods, other non-invasive tools may also be useful for diagnosing DR. Namely, response to flick response provides a stress to retinal vessels (that react differently in DR patients), while additional measurement of oxidation levels in retinal vasculature provides another potential way to distinguish non-DR from DR patients [3].

TPEF may have some interesting capacities and is now being developed as a technique that can safely be used in human eyes [51], making it possible to distinguish different retinoids in the retina, and in other settings, to quantify biomolecules in vivo [51]. This technique also allows for the imaging of optical sections in a non-invasive and safe manner, which can produce comparable results to those found using standard histopathology-sliced tissue sections [41,51,52,53].

## 3. RBP3/Two-Photon

### 3.1. RBP3

RBP3 has been linked to retinal development [30,114,115,116] and maintenance of retina integrity [35,110,117,118]. An ARVO communication presented a CRISPR knockout rat (-/-), and indicated that RBP3 is also necessary for the structural integrity of the outer segments of photoreceptors [119]. Although its best-known function is the shuttling of retinoids between photoreceptor and RPE cells supporting the visual cycle [14,36,120,121], it also transports essential lipids, such as docosahexaenoic acid (DHA) in the IPM [30,110,122,123], and offers photoprotection capacity for light-sensitive retinoids and free radical scavenging activity [102,110,111], which is important for modulating the IPM redox environment [102,110]. Thus, RBP3 plays a decisive role in maintaining the homeostasis of the outer retina [110].

Dysregulation of RBP3 has been implicated in several diseases [118] and results in the accumulation of retinal lipofuscin [30,110,124,125]. It is a primary cause of retinal degeneration in Abyssinian cats [30,110,126,127], and has been linked to myopia and retinal dystrophies in non-sense mutation in children [30,110,116,128], as well as one form of retinitis pigmentosa in a missense mutation [30,110,129,130].

Furthermore, and perhaps indicative of RBP3 as a marker of early disease states, the mouse model rd12, used in retinal degeneration studies, shows downregulation of the RBP3 protein [30,126,131]. Retinas from Streptozotocin-induced diabetic rats show decreased proteins levels of RBP3 [14,30], and lower mRNA levels in light-induced rat degeneration models [30,132]. Lower mRNA and protein levels of RBP3 have been reported in human retinas from diabetic donors compared to non-diabetic ones [30,31]. It is unclear if this is a cause or a consequence of retinal neurodegeneration in DR, but it has been established that low levels of RBP3 transcript and protein are related to poor disease prognostics, and thus is useful as an early DR detection tool [31].

#### 3.1.1. Historical

RBP3 is the most abundant soluble protein in the IMP, the subretinal space in vertebrate retinas, accounting for approximately 5% [30,31,35,36,118]; it was first reported by Adler and Severin in 1981 when it was extracted from bovine IPM. Shortly after, it was described as a glycoprotein that may be a transporter protein shuttling retinol between photoreceptors and RPE cells [133,134,135].

#### 3.1.2. Gene Duplication

RBP3 has a mass of approximately 135 kDa and is composed of four modules. Each is approximately 300 amino acids in length [30,136,137] with similar ligand binding; so one module can compensate for the potential malfunction of another [30,32,137]. The gene seems to have evolved from a quadruplication of an ancestral gene [137] and its expression is regulated by two essential transcription factors, the cone-rod-homeobox (CRX) and the orthodenticle homolog 2 (OTX2) [30,138,139].

#### 3.1.3. Ligand Binding

RBP3 is a crucial element for maintaining the visual cycle (Figure 2). The cycle is responsible for regenerating the chromophore 11-cis retinal to complex with the opsins of the photoreceptors after its photoisomerization and release in the form of all-trans retinal. Several enzymes are involved in the regeneration of the chromophore, and in vertebrates such enzymes are present in the photoreceptors and RPE cells. Thus, RBP3 provides a shuttling mechanism to bridge the two cell types across the IPM. In the canonical cycle, all-trans-retinal is reduced in the photoreceptor to all-trans-retinol and is transferred to the RPE where it gets esterified to all-trans-retinyl ester, converted to 11-cis retinol, and finally oxidized to 11-cis retinal which is transferred back to the photoreceptor. There is a second cycle called the cone-specific visual cycle in which the conversion of all-transretinol to 11-cis retinal occurs between the cone photoreceptors and the Muller cells [30,140,141], and it is also facilitated by RBP3 [30,142,143].

**Figure 5:**
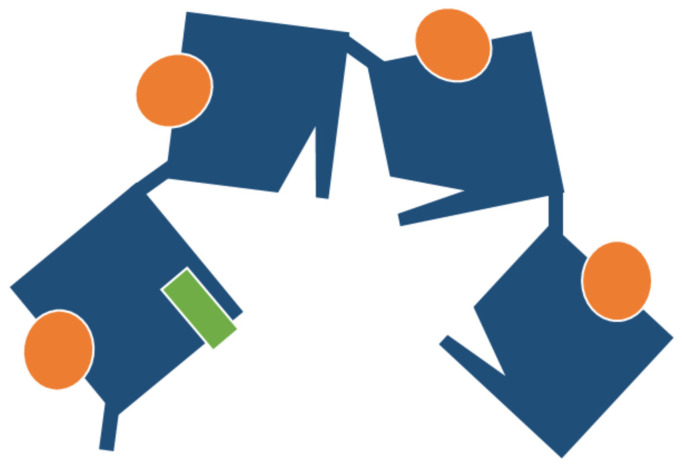
RBP3 depiction, with its four subdomains and binding sites for retinoids and fatty acids.

RBP3 binds many different retinoids with the following preferences: 11-cis-retinal > all-trans-retinol > all-trans retinal > 11-cis retinol [144,145]. It also has the ability to bind, albeit with lower affinities, to important lipids of photoreceptor membrane biosynthesis, such as the long-chain fatty acid docosahexaenoic acid (DHA) [30,146,147] or oleic acid [30,32,122,148] (Figure 5).

Due to its modular organization, a single RBP3 molecule can bind several ligands. If one module loses binding capacity or one of its binding sites is occupied by a labelled retinoid under our proposed approach, other sites would still support the protein function. Each module employs a “ββα-spiral fold” related to C-terminal processing proteases (CTPases) and to enoyl Co-A hydratase/isomerase (also known as crotonases) [32], and is composed of two qualitatively distinct domains [122]) with each having a hydrophobic cleft that can accommodate ligands [122], retinoids, and fatty acids [122,149,150]. Some sites (from particular modules) seem unavailable on the full-length protein with the tandem four RBP3 modules [150].

#### 3.1.4. Link to DR

RBP3 is the most significant soluble protein component in the IPM, is secreted from the photoreceptors, and is involved in the shuttling of retinoids between photoreceptors and RPE cells; and it has antioxidant properties [30,102]. It is assumed to have a half-life in IPM of approximately 11 h [2,35,151], being rapidly turned over through endocytosis in the RPE [35,110] (with a certain amount seemingly also internalized by the photoreceptors [152]); this rapid turnover may be a consequence of irreversible oxidation of RBP3 [102]. RBP3 protein distribution changes observably between dark and light conditions and is aligned with the changes in IPM volume due to hydration levels, as mentioned above [2,91,95,151,153,154,155,156,157,158,159]. Berkowitz speculates that the decrease of RBP3 leads to an increase in oxidative stress and dysfunctions in the dark-light IPM volume and RBP3 distribution [2].

Multiple lines of evidence link RBP3 with DR, where both diabetic patients and diabetic mice show reduced RBP3 protein levels [2,14,31,37], and patients with inherent elevated levels of RBP3 have some level of protection against DR [37]. The same study also offers evidence that intravitreous application of recombinantly produced RBP3 can inhibit VEGF-mediated retinal vascular permeability (RVP) in a dose-dependent manner, with the mRNA levels of *Vegfa* and *Il-6*, and of the VEGFA protein, being reduced after the RBP3 injections [37]. Increased ERG amplitudes of oscillatory potential (OP) 2, OP3, and B waves, also in diabetic mice, have been observed after intravitreal injection of recombinant RBP3 protein, as well as after RBP3 overexpression using a lentiviral vector injected subretinally, or when directed for expression in the photoreceptors by coupling it to the rhodopsin promotor [37].

Importantly, RBP3 is also present in the vitreous and its levels also decrease with the progression of DR, as has been observed in the retina [37]. The same study detected circulating RBP3 in the serum, but its levels are 1000- to 5000-fold lower (in the picomolar range) than in the vitreous (in the nanomolar range) in control patients without diabetes [37].

### 3.2. Two-Photon Excitation Fluorescence

**Figure 6:**
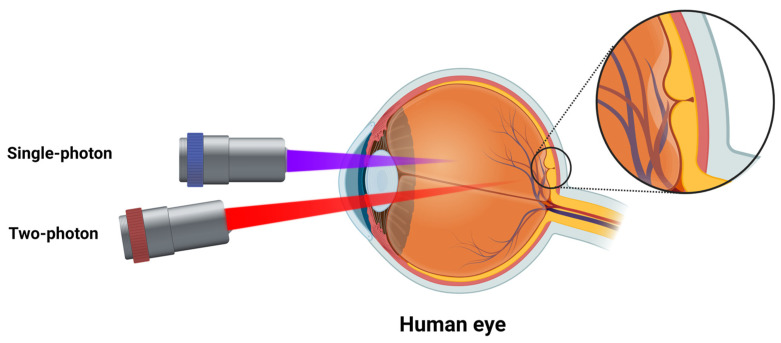
Illustration of one- and two-photon penetration power in retinal imaging.

Two-photon excitation fluorescence relies on the principle that two photons arrive at the same spot simultaneously, and that the recipient will experience an excitation of half the wavelength of the individual photons. Thus, one can achieve high energies by delivering a non-invasive, rather weak (but effective) laser beam in the infrared region [41,51,52]. This characteristic is particularly relevant for imaging of the eye, as intense high-energy wavelengths of light have detrimental effects and poorly penetrate the tissue, being absorbed by the anterior parts of the eye (sclera, cornea, lens) [53]. The penetration of light is further diminished in older people, making it even more difficult to use, as age is a major driver for pathological complications. Infrared light (IR) is much less affected by absorbance and scattering issues from biological materials, so better images are generated [29,51,53,160]. Another advantage of the two-photon method is the relatively large penetration power that allows the beam to cross the anterior part of the eye and the vitreous, and image the retina in the posterior part of the eye; the IR light beam keeps its shape through the ~250 μm retina thickness [51] (Figure 6). This advantage is particularly relevant in the context of DR, where the clinical and molecular manifestations occur in the retina and vasculature networks at the back of the eye. Currently, it is still not possible to acquire 3D volumes of the entire retina, but smaller 3D volumes enable imaging, for example, of ganglion cells and the IPM [51], or other subcellular localizations along the RPE thickness [52,53].

#### 3.2.1. Historical

TPEF was first used in ophthalmologic settings in 2004, when it was employed in the characterization of retinosomes (lipid droplets) that are rich in retinyl esters [52,161,162]. It has since been used to elucidate RPE storage compartments of vitamin A and their role in the visual cycle [29,161,162,163], as well as to examine the efficiency of the native visual cycle naturally and when it is modulated by test drugs [29,49,164,165]. Over the years, intensive research and development has aimed at reducing the IR light power needed to the point that it is now safe to use in the human retina [29,51,52,53].

#### 3.2.2. Clinical Applications

In ophthalmology, TPEF has been explored as a means of identifying the root causes that lead to retinal degeneration. Such degeneration has been measured using an array of imaging techniques (fundus photography, optical coherence tomography (OCT), scanning laser ophthalmoscopy (SLO)), but these do not provide cellular-level information and fail to identify the molecular starting points of the pathological conditions [51,52,53]. TPEF has proven to be particularly helpful in detecting defects in the retinoid visual cycle, which are linked with several diseases [51,166]. Typical visual cycle retinoids and important by-products, such as N-retinylidene-N-retinylethanolamine (A2E) and other components of lipofuscin, with typical maximal absorptions of less than 400 nm (when excited with single photons), allow for monitoring with TPEF imaging and, thus, the capacity to monitor retinal health [51,53]. Elsewhere, TPEF is steadily gaining popularity, particularly for the characterization of tumor microenvironments [41].

#### 3.2.3. Retinoids/Phasor

Coupling fluorescent lifetime with TP-imaging provides a powerful mechanism for further exploration of retinoids, early-stage imbalances, and the potential to detect diseases before macroscopic manifestations arise [52]. When measured data is displayed in Phasor plots, it is possible to distinguish between fluorophores that otherwise have overlapping spectra [42,167], as well as to distinguish monoexponentially decaying fluorophores (that are located in the universal semicircle line) from multiexponential ones (located inside the universal semicircle) [52,167].

#### 3.2.4. Possible Applications in a DR Context

As a proof of concept, our proposed approach still requires the injection of modified ligands or antibodies. However, we see this as a small price to pay, as it is similar to some of the current treatments and could have substantial impact on the prevention of vision loss. At first, antibodies and fab fragments may seem too bulky to reach the retina, but current anti-VEGF and anti-angiogenic therapies have revolutionised the way DR is treated, ranging from antibodies and fab fragments (e.g., Bevacizumab) down to small chemical molecules with molecular weights of ~600 g/mol (e.g., squalamine) [5]. Anti-inflammatory treatments use even smaller molecules that are more similar to the size of retinoid molecules, the actual ligand partner of RBP3.

## 4. Conclusions

This review argues for an in vivo biopsy-like quantification of RBP3 levels, serving as a DR biomarker and early diagnostic tool that would precede detections of visual impairment, vasculature changes, and even neuroretinal function impairment.

The eye has always been thought to be a window to the body (although the retina is one of the few tissues that cannot be biopsied [12]). Its direct observation can provide insight into the health condition, glucose levels, and many other variables. However, the composition of the retina and its opacity to many wavelengths of light, or the occasional need to use an ultraviolet regimen (UV) that is actually damaging and of low penetrance, impede some approaches. One option is to move to infrared technology, but here the readout can be masked by many of the eye’s natural compounds, which have overlapping emissions. The solution is to use TPEF that employs long wavelength light with deep penetration, and that can provide a combined excitation that will allow a “clean” emission signal. Indeed, TPEF has been used recently to detect retinoids in vivo.

Despite our proposed diagnostic method being micro-invasive (with the desired route being completely non-invasive), it is likely an acceptable trade-off due to the benefits that it could provide. Current treatments (besides strict metabolic control), such as intraocular drug delivery of steroids and anti-vascular endothelial growth factor (VEGF) [10] are definitely invasive.

Photocoagulation to treat diabetic retinopathy was first established in 1954, and 70 years later it is still the go-to treatment for the most severe cases of DR. Now that 100 years have passed since the introduction of insulin as a diabetic treatment, and with the advances in our understanding of the molecular mechanisms underlying DR and advances in development of imaging devices, we should at least consider and test the hypothesis that RBP3 can be measured in vivo, and that its quantification can be used as an indicator of the initial steps of DR development.
